# Hygieia model: development of a three-dimensional model for the standardized analysis of infection control protocols

**DOI:** 10.1007/s10389-023-01848-x

**Published:** 2023-02-20

**Authors:** Thomas Sakschewski, Claudia Winkelmann

**Affiliations:** 1 Berliner Hochschule für Technik, Luxemburger Straße 10, 13353 Berlin, Germany; 2 Alice-Salomon-Hochschule Berlin, Alice-Salomon-Platz 5, 12627 Berlin, Germany

**Keywords:** COVID-19 pandemic, Risk assessment, Event industry, Employees, Physical-mental-social health, Occupational insurance associations

## Abstract

**Aim:**

Until now, there have been no standardized guidelines for the content of infection control protocols. The aim of this research project is therefore to develop a standardized model for the evaluation and analysis of three dimensions: setting, protection targets, and precautions.

**Subject and methods:**

Events are part of social life and, as such, have a direct or indirect impact on the physical, mental, and social health of all involved groups (employees, artists, subcontractors, visitors, etc.). Valid infection control protocols for events must reduce the risk of infection in general, not only in a pandemic. A range of handouts and recommendations are available, mostly focusing on the visitors.

For the present study, a total of 46 infection control protocols for events, hosted in the period between 2020 and 2021 in Germany, were analyzed from June to December 2021. The infection control protocols provide what was needed to realize events.

**Results:**

For the first time, a standardized model, called the Hygieia model, is presented for the evaluation and analysis of three dimensions: setting, protection targets of the involved groups, and precautions. Taking all three dimensions into account enables the assessment of existing pandemic safety protocols as well as the development of valid protocols in terms of effectiveness and efficiency.

**Conclusion:**

The Hygieia model can be used for risk assessment of events from conferences to concerts, especially for infection prevention under pandemic conditions.

## Introduction

### Valid infection control protocols

Events are part of social life and, as such, have a direct or indirect impact on the bio-psychosocial health of all involved persons (employees, participants, visitors, etc.). In a pandemic, events must take into account and scale down the risk of infection by a suitable *infection control protocol* (ICP). Until now, there have been no standardized guidelines for the content of the ICP for events established by the Infection Protection Act (IPA); however, a range of handouts and recommendations have been issued by occupational insurance associations for employees and participants as well as for event organizers focusing on visitor safety. A standardized model for the evaluation and analysis of the dimensions *precautions*, *protection targets of the involved groups*, and *setting* is required. The consideration of all three dimensions allows for both the evaluation of existing ICPs and the development of valid ICPs under the aspects of effectiveness and efficiency.

### Typologization of events: the setting

For a hazard-oriented typologization of events, a range of influential factors must be studied. Some of these factors are a direct result of event planning and realization, and others can be deduced from the definition and prioritization of protection targets. Sakschewski and Paul (2017) cite the event organizer’s work experience and competence and the venue operator’s function, visitors in attendance, sociodemographic factors, and situational aspects attributed to the event experience, the type of event, the format of the venue, and the *Gestalt* (shape) of the venue site, meaning the spatial relationship between the scenery and the audience. Gestalt (shape) is a term borrowed from psychology. According to Gestalt psychology, human perception can be described as the ability to discern structures and principles of order in sensory impressions. In this context, the shape of an event means the spatial arrangement of scene and audience area as well as the movement of visitors in the event area and in relation to the surroundings. From the perspective of the authorities issuing the license, specifically factors such as venue site and attendance or the event organizer competence and work experience are studied (State Capital Munich 2015). For an evaluation of the infection risk for employees and participants, recommendations cited in the employer’s liability insurance association policies are crucial, which, if in doubt, result in a special risk assessment of the activities being planned.

The IPAs do not necessarily distinguish between types of events. This is evident, for example, in Article 20 of the State of Brandenburg’s Second Infection Protection Act for SARS-CoV-2 Control, bundling memorials, museums, exhibition halls, galleries, planetariums, archives, amusement parks, zoos, game halls, zoological and botanical gardens; theaters, concert and opera houses, cinemas, trade fairs, exhibitions, amusement arcades, casinos and betting offices; and fun and leisure pools, outdoor pools, saunas, thermal baths, and wellness centers (BRAVORS 2022).

Sources of risk for COVID-19 disease include infection by aerosol transmission, droplet infection, or smear infection (RKI 2021). The precautions laid down in the IPAs aiming to minimize the risk of infection are based on these sources of risk. Additionally, the Robert Koch-Institut (RKI) recommends that these precautions be consistently complied with, even post-vaccination (RKI 2022). The risk of aerosol transmission is higher at indoor events than at outdoor events. Here, there is a compelling distinction due to the event location, as is also evident in the infection control regulations. At standing-room-only outdoor or indoor events, there is an increased risk of droplet infection due to potential situations in which a physical distance in public cannot be maintained. In this case, it is much more problematic to communicate and monitor that visitors maintain a physical distance as required by technical and organizational precautions throughout the entire event (including entry and exit) with employees monitoring compliance.

The concept of *setting* is terminologically appropriate for the influential factors of space and visitors of the event. *Setting* includes both spatial and behavioral characteristics. Spatially speaking, a distinction is made between *outdoor and indoor events*. Behaviorally, the visitor *movement* is taken into account (Chittaro and Ieronutti 2004). It is easier to control the physical distance in events with assigned *seats* than in standing events. Taking into account the three statuses of visitor behavior to be expected and the two spatial principles of indoor and outdoor, what results for the influential factor of setting is a 2:3 matrix (Table 1).

**Table 1:** Characteristics of the influential factor setting

Setting		Behavior of visitors		
		Sitting	Moving	Standing
*R*oom (R)	*I*ndoor	R_ISI_	R_IM_	R_IST_
	*O*utdoor	R_OSI_	R_OM_	R_OST_

### Different protection targets

Safety planning for an event includes planning and controlling the safety of the event technology used, assessing and evaluating potential hazards to visitors, and planning and implementing safety precautions for employees and participants throughout set-up, rehearsals, the event, and disassembly (Sakschewski and Paul 2017). Employees fall directly within the scope of protection of labor law and, together with the participants, within the scope of protection of workplace safety (Winkelmann and Sakschewski 2021). Participants are indirect addressees of occupational health and safety. Participants are artists or employees of a hired subcontractor whose employers are obliged to cooperate in implementing workplace safety and health protection provisions and, going by the nature of the activities, to instruct each other and their employees about the risks to the safety and health of the employees associated with the work. However, freelancers such as performers or other freelance participants at an event are not employees within the meaning of the Occupational Health and Safety Act (ArbSchG 2020). The scope of protection of status of employees and participants—which is based on the activity and not on the status of employment—can also be deduced from the scope of application of the precautionary regulations laid down by the employers' liability insurance association. They apply to the stage and performance area of event venues and the production and performance area of production facilities without making additional distinctions between the groups of people working here (DGUV 2013).

On the one hand, visitors have a right to physical integrity and, on the other hand, a right to the free development of their personality in Germany (Basic Law 2020). The organizer, that is to say, the legal or natural person authorized and competent to host and manage the event, is responsible for safety at the event, including visitor safety throughout the event, and for compliance with the regulations, including compliance with the latest applicable IPA, consistent with German venues and events regulations (Klode 2020). Accordingly, organizers are obliged to ensure that the visitor health and life are protected and not put at risk by the type, duration, or location of the event. At the same time, it must be planned and considered that individuals can and may, of their own free will, expose themselves to a situation with a higher risk potential and as an expression of the free development of their personality. The house rules for event venues and temporarily booked event locations provide the applicable legal framework. Such being the case, in comparison to employees, there is no direct authority to issue instructions, and in comparison to participants, there is no indirect supervisory power and information obligation. Safety protocols are planned and implemented in order to keep risks for visitors at bay.

Recommendations and guides on the planning and realization of events are available from authorities, organizations, and initiatives, which refer to infection prevention of employees and, in some cases, of participants as well (AGVSa 2020; VBG 2021). Germany’s state-specific IPAs as well as handouts of the competent administrations and other initiatives (Berlin Hygienic Framework Concept 2022; AGVSb 2020) provide guidance for visitor safety at events. The interaction of the involved groups—employees, participants, and visitors—is always considered when there is person-to-person visitor contact throughout the event. In the pandemic safety protocols, precautions are aligned with the protection targets. Consequently, employees fulfill a double function when it comes to infection control. They are both the target of the planned precautions and the involved groups in the implementation of precautions when handling visitors and participants. Employees are required to monitor compliance with these precautions and to step in if visitors fail to comply.

The planned precautions can be split into technical, organizational, and personal. *Technical precautions* are those that minimize a hazard at the source. *Organizational precautions* eliminate a risk by defining procedures, and *personal precautions* provide individual protection against a hazard. Applicable law allows that technical precautions are to be preferred to organizational precautions and the latter to personal precautions. The requirements, such as maintaining a physical distance of 1.50 m at all times, can be implemented, for example, by technical precautions such as blocking off seating areas, or by organizational precautions such as downsized attendance allowed to simultaneously enter the site, or designated pathways (IPA 2022).

### Three-dimensional evaluation model for the analysis of infection control protocols

ICPs are required in both the federal state-specific and nationwide levels in the IPA. Protocols should specify precautions for

1. controlling the entry,
2. recording the chains of infection (by recording personal data using an app, paper documentation, etc.),
3. minimizing the risk of infection,
4. defining safety protocols,
5. defining how employees, participants, and visitors will be informed about these precautions, and
6. monitoring the implementation of precautions (IPA 2022).

Entry control: The ICP must specify how entry is to be restricted for the attendance allowed, which type of certificates are considered a valid proof of status (vaccinated, recovered, tested) and what format (analog, digital), and how to respond to disruptions. Technical/structural precautions (separation, early entry) and organizational precautions (number of entry gates and staffing) must be taken into account.

Minimizing potential centers of infection by rapid detection of chains of infection: This requires end-to-end documentation of personal data of employees, participants, and visitors throughout all stages of the event (set-up, rehearsals, entry, performance, exit, and assembly). Under Section 2, Article 16 of the Infection Control Act, personal data include the following: surname and first name, gender, date of birth, address of the main residence or usual address and, if different, current address of the person concerned and, if available, telephone number and e-mail address. The federal states’ IPAs and SARS-CoV-2 control govern the filing of personal data in different ways. What they have in common is the event organizer’s obligation to record personal data, to save them for the span of four weeks in a manner protected from access by third parties, and to delete them after four weeks have passed (digital documentation) or to destroy it (analog documentation). The data have to be handed over to the competent authority on request in cases where it is established that a person was ill, suspected of being ill, infectious, or a carrier as specified in the Infection Control Act applicable at the time of the event, visit, or use of the service. The data may be used exclusively for contact tracing consistent with infection control legislation, that is to say, only with express permission for advertising campaigns and information about other events. Which data are to be filed, however, varies between the state-specific IPAs.

Minimizing the risk of infection: This included a comprehensive overview of all technical-structural, organizational, and personnel precautions (TOP) pre-event, mid-event, and post-event (entry, event, end), with specific consideration given to attendance, visitor loads, ventilation, social distance requirements, and mouth-nose coverage. To this end, the state-specific IPA needs to be complied with, with municipalities authorized to add their own regulations to these codes.

ICP: The ICP for events lays down precautions for cleaning or disinfecting repeatedly used spaces, providing disinfection facilities for event-goers at the entrance area, and safety precautions for employees and participants, especially for person-to-person visitor contact. The obligation to prepare a health protocol is primarily enshrined in the federal states’ IPAs or Corona or SARS-CoV-2 control. Additionally, municipal bodies or other organizations such as the regional chambers of commerce and industry issue recommendations.

Information and control: Officials of the competent authority and the public health department are authorized to carry out investigations and to monitor the required precautions. A person responsible for on-site health and safety should expect the precautions laid down in the protocol to be checked at any time, and must ensure that employees, participants, and visitors comply with the required precautions in order, on the one hand, not to violate the duty of care and, on the other hand, not to risk regulatory penalties or to risk the event being cancelled. This requires, on the one hand, visitor compliance supported by explanations and information, and, on the other hand, competent and authorized members of staff making sure that precautions are observed, and intervening if they are violated (Winkelmann and Sakschewski 2021).

## Methods

**Fig. 1:**
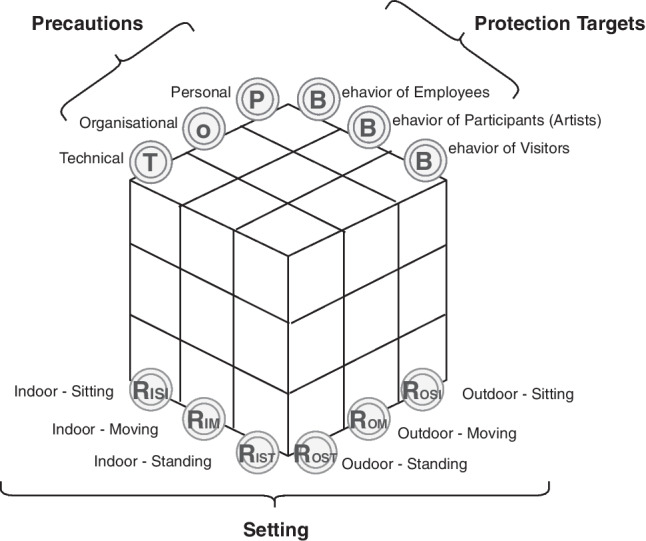
Hygieia model for analyzing and evaluating infection control protocols

In order to work out a model for analysis and as a preliminary matter, the events need to be typologized. To this end, the event setting as well as the attendance are considered, since no risk propensity can be directly deduced from the total attendance (AGVSa 2020). What is relevant, however, is the setting, that is to say, an event’s spatial-behavioral characteristics. Here, influential factors are space and visitor behavior that is to be anticipated depending on the Gestalt (shape) and type of event. The evaluation matrix thus maps out a scheme that departs from prior evaluation practice. In the IPAs, maximum attendance capacities for outdoor and indoor events have been set and continue to be set. An ICP is addressed to visitors as protection targets, to employees in the capacity of supervisors as protection targets, and to participants in the capacity of performers as protection targets. Going by the hierarchy of precautions known from occupational health and safety, precautions cited in the ICPs can be split into technical precautions (planning, assembly, ventilation, interior design), organizational pre-event, mid-event, and post-event precautions, and personal precautions primarily aimed at visitors, employees, and participants. This results in a three-dimensional model for the analysis of ICPs, in which the involved groups are to be considered as protection targets, precautions are subdivided according to the technical-organizational-personal (TOP) principle, and events are to be differentiated according to the setting (Fig. 1).

Existing ICPs were analyzed consistent with the typologization of the events going by the setting, the aim being to study whether differences in the precautions could be identified in relation to the protection targets of the involved groups and the setting.

For the study, a total number of 46 ICPs for events hosted in the period between 2020 and 2021 in Germany have been analyzed from June to December 2021 (Table 2).

**Table 2:** Number of infection control protocols examined by federal state

Federal state	Number
Baden-Wuerttemberg	8
Bavaria	3
Berlin	16
Brandenburg	5
Hamburg	1
Hesse	1
Lower Saxony	5
North Rhine-Westphalia	4
Saxony	2
Saxony-Anhalt	1

The ICPs provide what is needed to realize the events. They are mandatory for event organizers in accordance with the state-specific IPAs. Submission to or review by the competent health authorities or state health bodies is at the discretion of the authority and, in practice, depends on the expected attendance, the incidence rate, familiarity with the event organizer or event, and the latest available IPA. Not all of the 46 pandemic safety concepts for events included in the sample were realized at the originally scheduled time. In some cases, they were rescheduled or canceled due to modifications to the IPAs.

The ICPs take into account the requirements of the infection control regulations valid at the time of the event being hosted as well as state-specific procedural regulations on maximum attendance, precautions to be taken, and the entry situation. Depending on the event type or event schedule, recommendations and instructions for employees are also incorporated. In venues with a high number of technical and artistic staff such as theaters and opera houses, different conditions in production and workshop spaces, on stage, in the dressing room, in the make-up room, in the orchestra pit, or in lighting are documented in the company’s ICP, including specific precautions for different departments and their workplace conditions in addition to the general regulations.

In order to process the pool of ICPs, a quantified analysis of qualitative data was carried out as a secondary data analysis. In this case, the frequency of relevant words from texts is analyzed (Kuckartz 2014; Witzel et al. 2008). The procedure here is to count the frequency of words in relation to single precautions and to relate it to the total number of words (Mayring 2010). The total number of words is the total word count, excluding indexes, titles, footnotes, and headers. With this as a premise, the text sections and words in the entire text are categorized going by technical, organizational, and personal precautions and analyzed by word count. In addition, single precautions are quantified as well. Subsequently, the technical, organizational, and personal precautions are attributed to the involved groups (visitors, employees, participants) going by the purpose and the protection target of the precautions, and analyzed in terms of word count.

As a preliminary matter and for the purpose of the analysis, the special ICPs are categorized going by event settings (Fig. 1, Table 1).

## Results

### Relevant words indoor

For an in-depth analysis of the event settings, the ICPs were examined for precautions taken in the technical, organizational, and personal areas. In doing so, codes are set up for single precautions that are intended to minimize the risk of infection by the coronavirus, with the word frequencies defining these precautions being counted. Synonyms and different spellings such as “spit shield, “sneeze guard,” “cough shield,” or “droplet guard” have been consolidated into one code. The codes are semantically deduced from the specifications in the latest available ICPs.

**Fig. 2:**
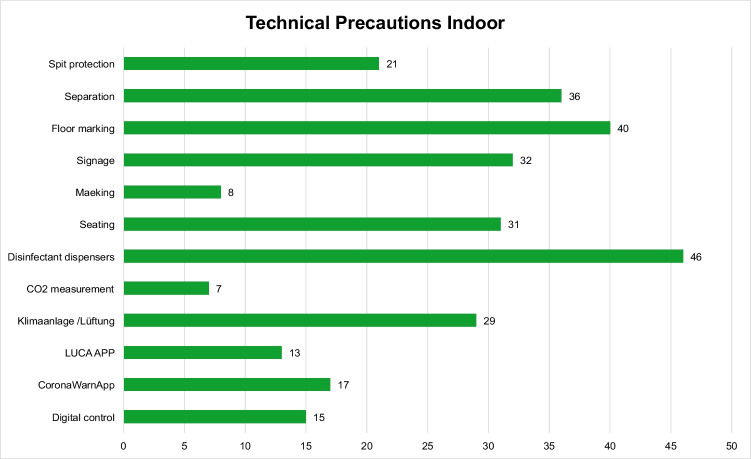
Number of single technical precautions in infection control protocols at indoor events

For the technical precautions, 12 codes have been set up for single precautions (Fig. 2). These are temporary structural modifications of varying scope on the venue site or in the buildings hosting the event, but also include apps for identity tracking (Luca App—Luca is an app tracking chains of infection of SARS-CoV-2 by contactless registration) or checking the vaccination status (Corona-Warn-App). With 46 cases, providing disinfectant dispensers, especially in entrance and sanitary areas, is cited most frequently among the technical precautions set out by the ICPs. The technical precautions for compliance with the distance requirements, such as separation of pathways and premises (36 cases), floor markings (40 cases), and seating adapted to the physical distance of 1.50 m (31 cases) are defined very frequently in the protocols as well. For employees who have person-to-person visitor contact, spit guards are often used for their own protection (31 cases). Another repeatedly cited technical precaution (29 cases) in confined spaces is adaptation, which is the conversion from recirculated air to 100% supply air, or modernized air conditioning and ventilation systems to provide fresh air. In some cases, CO2 sensors are utilized in order to measure the air quality (7 cases). Both precautions have been documented separately, the reason being that CO2 sensors can also be a possible precaution without upgrading or adapting the ventilation system. For the purpose of digital tracing of SARS-CoV-2 infection chains, the Luca App (Luca is an app tracking chains of infection of SARS-CoV-2 by contactless registration) is cited 13 times for indoor events.

For the organizational precautions, 18 different codes of single precautions have been set up to minimize the risk of infection with the help of organizational tools at events. Among the organizational precautions, cleaning and disinfection of contact surfaces and materials is mentioned most frequently (135 cases). Beyond that, registration and visitor contact tracing is mentioned repeatedly (72 cases) in the ICPs. Following modifications in the IPAs, event organizers and event agencies have to control the entry situation for visitors. The 2G rule (2G means proof obligation and admission only for recovered and vaccinated persons) is mentioned with 50 cases in the ICPs. For employees, the 3G rule (3G means detection obligation and admission for recovered and vaccinated or tested persons by rapid test) as provided in occupational health and safety regulations is cited. The testing regime is specified depending on special entry regulations. To give an example, visitors must show a daily antigen rapid test from an authorized testing site. For employees, rapid antigen tests are often provided by the facility. Employees are required to be tested at regular intervals. Participants, especially performers from abroad, are often required to show a valid polymerase chain reaction (PCR) test. These precautions are compiled under testing regimes (36 cases). To avoid densely packed entrance areas, lobbies, and other places outside the actual event site, pathways are designed as one-way paths in some ICPs (20 cases) and downsized attendance is allowed to simultaneously enter the site (39 cases). For employees, work hours and lunch breaks, as well as work group assignments, are scheduled to eliminate overlap and minimize the infection risk among employees and participants. This precaution was found in 49 locations. The citation of an appointed sanitation officer by name was found in as little as 24 locations, despite it being an obligation imposed by the IPA. Communication tasks cover a greater portion, which can be pooled under communication (24 cases), references (39 cases), or recommendations (28 cases). In those cases where the ventilation system could not be technically upgraded at the venue site, or as a supplemental organizational precaution, ventilation, with 41 cases, is number five on the list of the most cited organizational precautions in the indoor ICPs (Sakschewski and Winkelmann 2022).

**Fig. 3:**
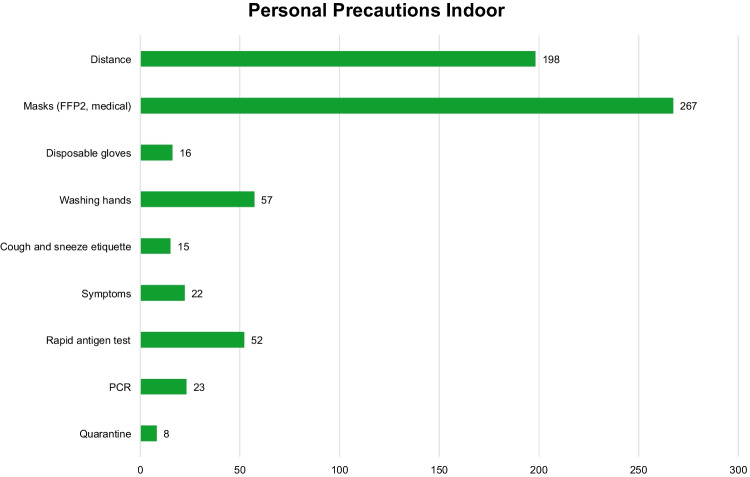
Number of single personal precautions in the infection control protocols in the indoor area

For the personal precautions, nine different codes with several single precautions were identified. It is important that the single precautions are not to be taken by organizers or their employees and contracted service providers, but directly by all involved persons (Fig. 3). If, at an indoor event, the minimum physical distance of 1.5 m cannot be maintained, and if it is a standing-room-only affair, wearing a nose-mouth cover is a must. Such being the case, this is also the most frequently defined personal precaution, with 267 cases. Compliance with the distance requirement of at least 1.5 m is cited in the ICPs as the second most frequent precaution (198 cases). Another important precaution for all agents is disinfecting and washing hands. For employees with person-to-person visitor contact, wearing disposable gloves is mandatory (16 cases). Visitors are advised to behave responsibly if they have symptoms and to stay away from the event (22 cases) if that is the case. Employees are required to take rapid antigen tests (52 cases) or PCR tests (23 cases) and to quarantine if they have symptoms and to inform their employer. Additional transmission precautions cited in the ICPs are hand hygiene (57 cases) and cough and sneeze etiquette (15 cases).

### Relevant words outdoor

For an in-depth analysis of the outdoor-event setting, the texts were examined for precautions in the technical, organizational, and personal areas. In doing so, codes are set up for single precautions that are intended to minimize the risk of infection by the coronavirus, with the word frequencies defining these precautions being counted. Synonyms and different spellings such as “spit shield,” “sneeze guard,” “cough shield,” or “droplet guard” have been consolidated into one code. The codes are semantically deduced from the specifications in the latest available ICPs.

**Fig. 4:**
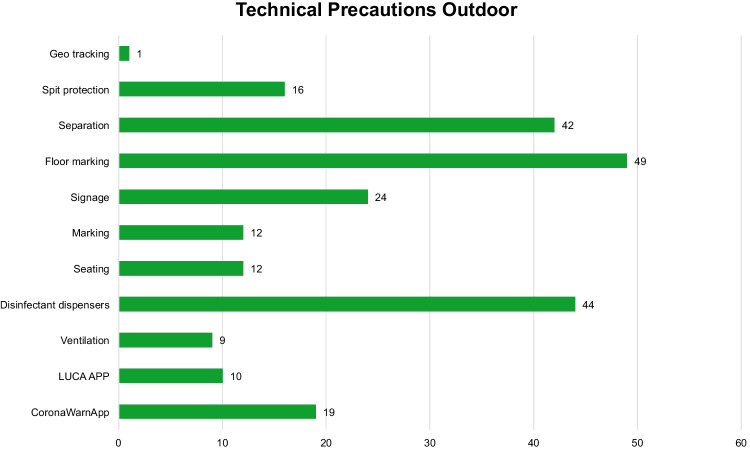
Number of single technical precautions in the infection control protocols in the outdoor area

For the technical precautions, ten codes aimed at minimizing infection rates have been set up. In substance, these are structural modifications on the venue site (Fig. 4). Providing floor markings to comply with social distance requirements (49 cases), separating pathways and rooms (42 cases), and providing disinfectant dispensers (44 cases) are requirements that are mentioned most frequently in the ICPs. Seating in a public area is cited exclusively in the ICPs according to room-outdoor sitting. For employees having person-to-person visitor contact, spit protection is used for their own protection, with 16 cases being cited. For digital tracing of SARS-CoV-2 infection chains, the Luca App is mentioned (10 cases). The Corona-Warn-App is mentioned more often than the Luca app for check-in at outdoor events (19 cases). Luca is an app tracking chains of infection of SARS-CoV-2 by contactless registration.

For the organizational precautions, 17 codes have been set up as single precautions to minimize the risk of infection at events. Among the organizational precautions, cleaning and disinfection of contact surfaces and materials is mentioned most frequently (93 cases). Instructions are defined for employees on how to divide up into work groups and on working hours during assembly and disassembly (61 cases). In addition, employees are regularly instructed in infection control and hygiene precautions, which can be compiled under training (30 cases). For each event, a sanitation officer has to be appointed. The capacity is also listed to a lesser extent compared to indoor protocols (23 cases). Communication between visitors, employees, and the event organizers as well as instructions on specific rules of conduct are frequently (34 cases) cited as organizational precautions. Beyond that, visitor registration and contact tracing are important elements in ICPs (53 cases). Since outdoor events were primarily hosted in the summer months and the analyzed ICPs date from 2020 and 2021, only the 3G rule (3G means detection obligation and admission for recovered and vaccinated or tested persons by rapid test) governed by the IPAs were applied for these event settings (13 cases). Visitors, employees, and participants, as an example, are required to show a daily updated rapid antigen test from an authorized testing site. Participants, especially performers from abroad, often are required to show a valid PCR test, which is compiled under “testing regime” (23 cases). To avoid densely packed entrance areas, pathways are designed as one-way paths (25 cases), and downsized attendance is allowed to simultaneously enter the site in 30 cases (Sakschewski and Winkelmann 2022).

**Fig. 5:**
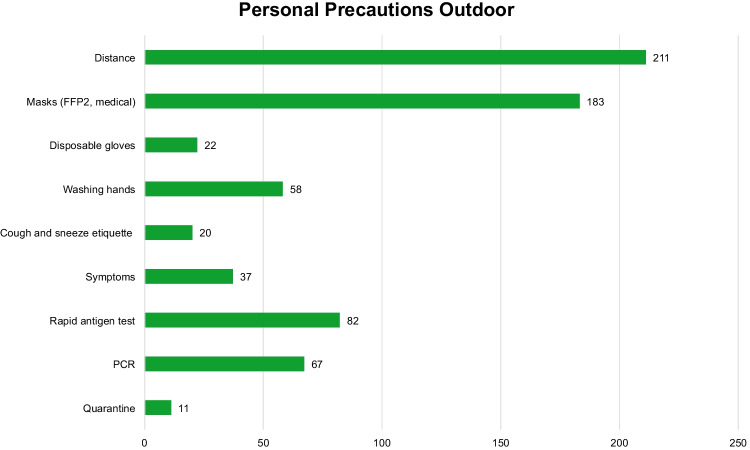
Number of single personal precautions in the infection control protocols in the outdoor area

For the personal precautions, nine different codes were set up for the outdoor settings aiming to protect individuals by personal precautions (Fig. 5). Cited most frequently in the ICPs in the outdoor area (211 cases) is compliance with the physical distance requirements of at least 1.5 m; wearing a nose-mouth cover or, synonymously, FFP2-mask, surgical mask, or mouth-nose protection is obligatory if the physical distance requirements cannot be met and, as a consequence, is mentioned frequently in the present ICPs (183 cases). Disinfecting and washing hands is another important precaution for all involved groups (58 cases). For employees with person-to-person visitor contact, wearing disposable gloves is mandatory (22 cases). Visitors are advised to behave responsibly if they have symptoms of illness and not to attend the event (37 cases). Employees are required to take rapid antigen tests (82 cases) or PCR tests (67 cases).

## Conclusion

When it comes to content, handling, and specification of protection targets, ICPs are not governed by an IPA, by regulations, or by other policies. However, they are mandatory for hosting events. That being the case, in the period between 2020 and 2021, event organizers or authorized third parties prepared ICPs within their scope and, as required, submitted them to the health authorities for review. On the basis of existing typologizations of events specified in the specialist literature and an analysis and evaluation of risk factors to be taken into account, it was possible to work out a standardized three-dimensional model in which types of events are studied as settings according to space and use of space by setting, protection targets of the involved groups, and precautions. Subsequently, the model’s applicability and selectivity were empirically studied by evaluating 46 ICPs.

What can be confirmed is that in the ICPs, precautions are broken down into technical, organizational, and personal precautions addressing the different protection targets of the involved groups. It can be assumed that the ICPs are of high operational relevance and are not to be understood as precautionary pseudo-strategies put on paper, but more so to be on-site guidelines. To what extent the implementation is also complied with and controlled depends on multiple factors that are to be considered in isolation from the three-dimensional model (Winkelmann and Sakschewski 2021). Regardless of the setting, organizational precautions are defined in the most detail, which can be attributed to the nature of the precautions and the purpose of the ICPs, because these are directly incorporated into staff briefings. Accordingly, the implementation is defined with greater precision here than is the case for technical precautions, which are repeatedly implemented by hired third parties or subcontractors, and then require further performance specifications that are not part of the ICP. The personal precautions are also not necessarily defined in the ICP but are amplified by specifications in the sense of occupational health and safety instructions for the involved groups, employees, and participants (security guards, artists, etc.).

The feasibility of working out a typology with the help of the model is also empirically provable with regard to the setting. Although it can be verified that organizational precautions predominate in both indoor and outdoor settings, more personal precautions are taken in the ICPs in indoor settings due to the higher risk of infection by aerosol transmission in confined spaces. In order to comply with these personal precautions, appropriate technical precautions are also defined to scale down aerosol transmission and to comply with social distance requirements. The total volume of ICPs varies greatly and seems to depend on the setting (maximum value: 4565 R_OST_, minimum value: 1050 R_OSI_). As it can be assumed that, going by the maximum attendance allowed at the time of the event, the event in the R_OST_ setting also has had larger attendance, so the maximum permitted attendance combined with risk propensity may be effective in this case. The partially low volume of ICPs available for evaluation fails to allow for a more precise statement here.

From the semantic content analysis, it was possible to identify different single precautions between indoor and outdoor settings and to count them with varying frequency of cases. It is obvious that ventilation and air conditioning are single precautions in indoor settings; however, physical distance requirements are mentioned with high case counts in both indoor and outdoor events. Disinfection dispensers are repeatedly mentioned indoors and outdoors as well. They are a visible sign of the safety task being fulfilled, they are recommended in handouts issued by the professional associations (VBG 2021), and documents issued by the relevant associations and administrations (SenKE 2022), yet they have only a minor immediate benefit, the reason being that the SARS-CoV-2 virus spreads mainly via airborne transmission (droplets and aerosols). What is limiting the analysis and evaluation of coded single precautions is the highly dynamic nature of recommended and implementable precautions throughout the study period. Far-reaching testing regimes were yet to be made available in 2020, the Corona Warn App or Luca App (Luca is an app tracking chains of infection of SARS-CoV-2 by contactless registration) gained public acceptance only after the evaluation period, and proof of vaccination status only became relevant in the second half of 2021 following amendments to the IPA.

The ICPs primarily address visitors with technical, organizational, and personal precautions, with employees cited to a much lesser extent, although they are at the same time protection targets and those implementing and monitoring the precautions for visitors. Participants (e.g., artists) receive little consideration. They are not under protection of occupational health and safety within the event responsibility of the organizer. Because they are marginally employed by a third party or they are freelancers, they are not given sufficient consideration and consequently pose a risk for the visitors and employees, on one hand, and are themselves at elevated risk of infection, on the other.

With that said, the three-dimensional approach in Fig. 1 provides a standardized model for ICPs, called the *Hygieia model*, which could be confirmed on the basis of the empirical evaluation of 46 ICPs. The next step is to work out a sample typology for the six different settings.

## Data Availability

Data available from first author
